# Case Report: PRES-Like Syndrome With Reversible Cortical Blindness Associated With Essential Thrombocythemia

**DOI:** 10.3389/fneur.2021.743165

**Published:** 2021-09-17

**Authors:** Ying Li, Yuanfeng Miao, Meng Yu, Ying Zhu, Zeyin Liang, Zhaoxia Wang, Qing Peng

**Affiliations:** ^1^Department of Neurology, Peking University First Hospital, Beijing, China; ^2^Department of Radiology, Peking University First Hospital, Beijing, China; ^3^Department of Hematology, Peking University First Hospital, Beijing, China

**Keywords:** posterior reversible encephalopathy syndrome, reversible cortical blindness, essential thrombocythemia, periodic triphasic waves, palinopsia

## Abstract

**Background:** There are few reported cases of posterior reversible encephalopathy syndrome (PRES) combined with essential thrombocythemia (ET). We report a case of PRES-like syndrome in ET.

**Case Report:** A 60-year-old man with a history of hypertension and thrombocythemia presented with progressive visual loss after waking up; and neurological examination showed pupils were 3 mm and equally reactive to light, which suggested cortical blindness. Brain magnetic resonance imaging (MRI) revealed restricted diffusion in diffusion-weighted imaging (DWI) in the bilateral parietal and occipital lobes. Routine blood tests revealed a platelet count of 1,044 × 10^9^/L. ET was diagnosed after exclusion of other causes. Electroencephalography (EEG) showed periodic triphasic waves in the occipital region. The lesions of the parietal and occipital lobes on MRI and periodic triphasic waves of EEG disappeared quickly, and patient's vision returned to normal after the treatment with hydroxyurea and sodium bicarbonate. The patient experienced hallucinatory palinopsia during the recovery of vision.

**Conclusion:** ET may be a risk factor for PRES.

## Introduction

Posterior reversible encephalopathy syndrome (PRES) is characterized by acute neurological symptoms (seizures, encephalopathy, headache, and visual disturbances) in the setting of renal failure, blood pressure fluctuations, cytotoxic drugs, autoimmune disorders, and preeclampsia or eclampsia (1, 2). Characteristic radiographic findings include vasogenic edema in the bilateral subcortical regions that resolve within days or weeks. PRES is generally reversible, both radiographically and clinically, and has a favorable prognosis (1, 2).

Essential thrombocythemia (ET) is a chronic myeloid neoplasm characterized by symptoms from small or large vessel occlusion and hemorrhage (3, 4). In this report, we describe a PRES-like patient who developed bilateral reversible cortical blindness combined with ET.

## Case Report

**Figure 1:**
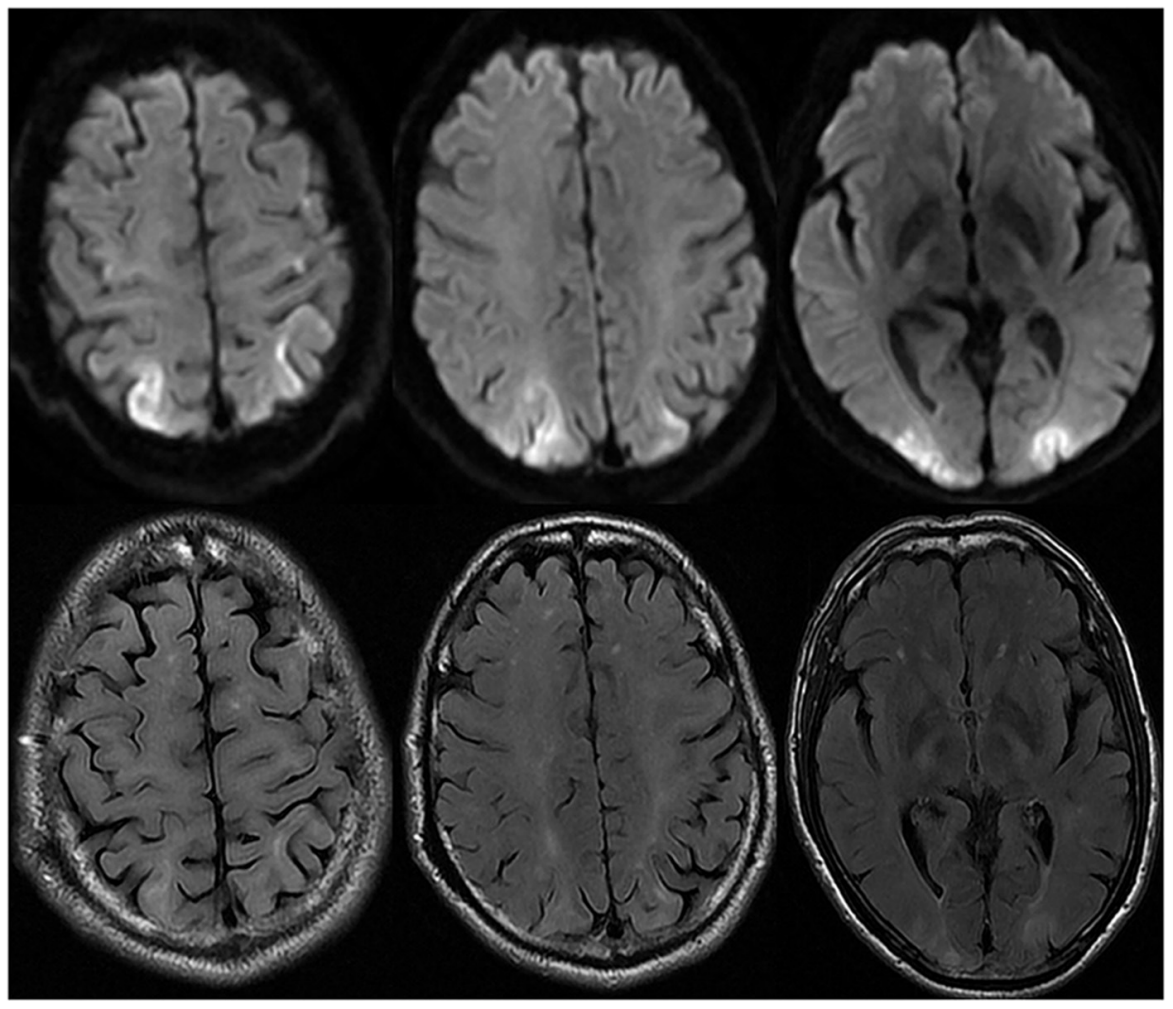
Brain magnetic resonance imaging on the second day after onset. Brain MRI showed diffusion restriction in diffusion-weighted imaging (DWI) and hyperintensity of fluid-attenuated inversion recovery (FLAIR) in the bilateral parieto-occipital lobe on the second day after onset.

A 60-year-old man with a history of hypertension presented to the emergency with sudden visual loss for 7 h after waking up. Thrombocythemia was found in routine physical examination 7 years ago, and the platelet count was between 400 and 600 × 10^9^/L. The annual physical examination showed that the liver function, renal function, and abdominal ultrasound were normal. He took aspirin 100 mg/day regularly, but aspirin was stopped 1 week before onset. Platelet count was found to be elevated to 1,044 × 10^9^/L after admission. The patient took telmisartan, and the blood pressure was about 120/80 mmHg in the past. The patient denied the history of alcoholism. Neurological examination revealed that his visual acuity was severely impaired on both eyes, and he could only recognize waving hands. His pupils were 3 mm and equally reactive to light. Blood pressure was 128/84 mmHg. Electrocardiogram showed atrial fibrillation. Urinalysis, liver function, renal function, and ophthalmic examination were normal. Considering that acute cerebrovascular disease could not be excluded, the patient underwent head non-contrast computed tomography and computed tomography angiography (CTA) and computed tomography perfusion (CTP) imaging, which showed no vascular stenosis or cerebral hypoperfusion. After 3 h, the visual acuity of the patient deteriorated, and there was only light perception. There was no obvious change in the second CT scan, and residual contrast agent was found in the venous sinus. However, brain magnetic resonance venography (MRV) showed no venous sinus thrombosis. Brain magnetic resonance imaging (MRI) on the second day after onset (Figure 1) revealed restricted diffusion in diffusion-weighted imaging (DWI) and reduced apparent diffusion coefficient (ADC) in the bilateral parieto-occipital lobes. A bone marrow biopsy showed myeloid granulocytic and megakaryocytic hyperplasia with myelofibrosis. Genetic analysis showed DNMT3A W860R mutation. No mutation was found in *JAK2, CALR*, and *MPL* genes. Contrast-enhanced MRI and CT of abdomen revealed stenosis of the trunk and cavernous transformation of the portal vein (CTPV), splenomegaly, splenorenal shunt, and gastric varices. C-reactive protein, erythrocyte sedimentation rate, and hemoglobin were normal. Essential thrombocytosis was diagnosed by the hematologist after excluding other causes. The patient developed left upper limb shaking similar to myoclonic seizure within 2 days after the onset of the disease, and then it was relieved spontaneously. Electroencephalography (EEG) showed periodic triphasic waves in the occipital region.

**Figure 2:**
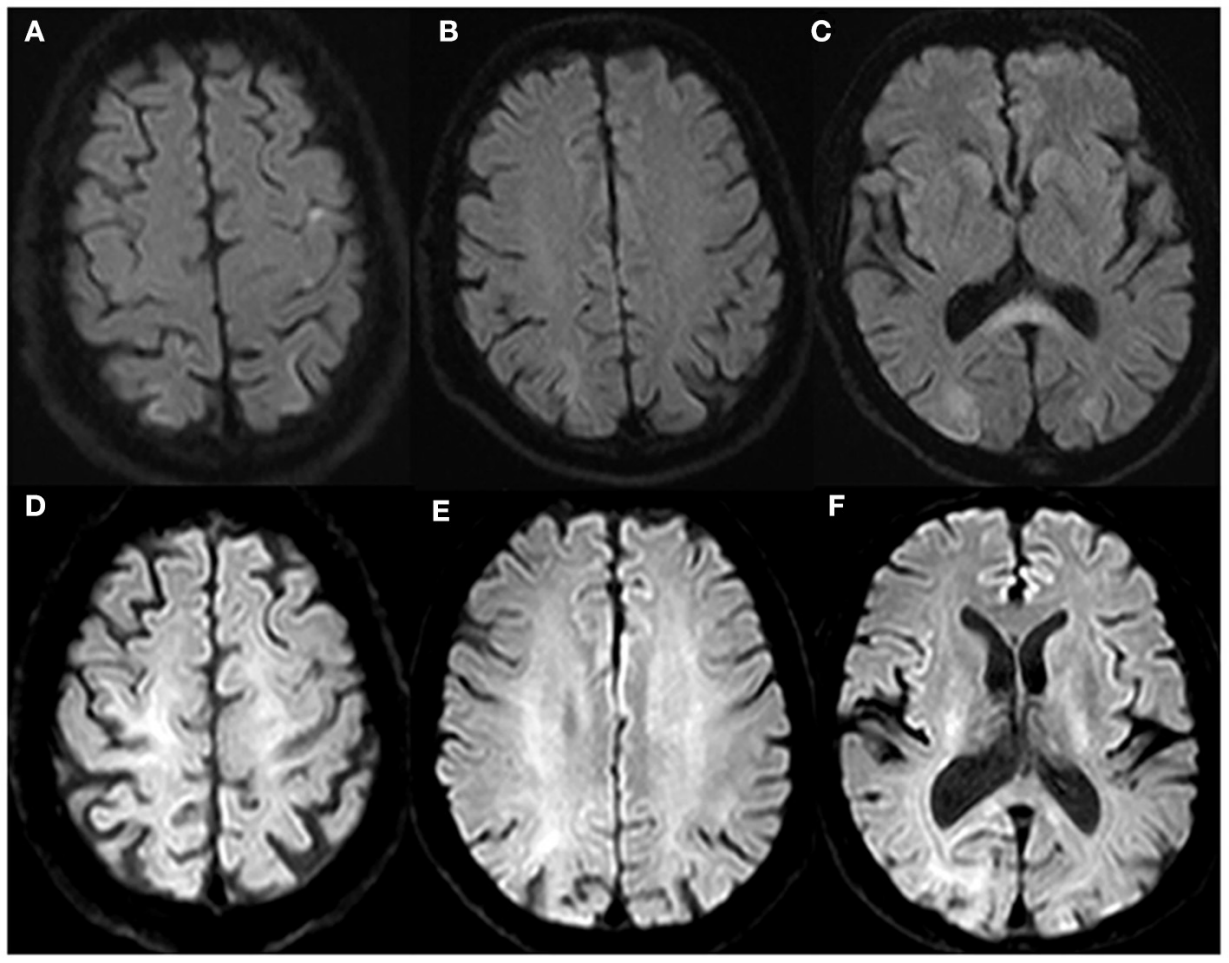
Brain magnetic resonance imaging in follow-up. Brain MRI showed new diffusion-weighted imaging (DWI) diffusion restriction in the splenium of the corpus callosum, and diffusion restriction of the parietal and occipital lobes was lower than before on the eighth day of onset **(A–C)**. Brain MRI revealed the lesions in the bilateral parieto-occipital lobe and splenium of the corpus callosum disappeared, leaving slight atrophy of the bilateral occipital lobe after 4 months **(D–F)**.

The patient was treated with hydroxyurea and sodium bicarbonate and supportive treatments. On the second day, the patient's vision recovered slightly and had palinoptic afterimages manifesting incorporation of one part of a previously viewed object or scene on to the visual image of a contemporaneously viewed second object or scene. The patient's vision returned to normal on the fifth day of onset. The platelet count decreased to 620 to 760 × 10^9^/L. On the eighth day of onset, the second brain MRI (Figures 2A–C) showed new restricted diffusion in the splenium of the corpus callosum, and hyperintense signal of the parietal and occipital lobes in DWI was lower than before. A diagnosis of PRES was considered. The EEG reexamined 2 weeks after the onset revealed that the triphasic waves disappeared. The patient took aspirin after discharge. Four months later, brain MRI (Figures 2D–F) showed that the abnormal signals in the bilateral parieto-occipital lobes and the splenium of corpus callosum disappeared, leaving slight atrophy of the bilateral occipital lobe with microbleeds.

## Discussion

To our knowledge, this report is the second to document a case of PRES and ET (5). Brain MRI in our patient showed restricted diffusion in DWI and reduced ADC indicating cytotoxic edema, which is consistent with ischemic infarction and different from vasogenic edema of typical PRES. Furthermore, the patient has a stroke-like onset; and several cerebrovascular disease risk factors such as ET, atrial fibrillation and hypertension, and ischemic infarction should be considered. However, no enhancement on enhanced brain MRI, the appearance of lesions in the splenium of the corpus callosum, and the regression of abnormal signals in the occipito-parietal lobes after treatment further support the diagnosis of PRES. According to the literatures, restricted diffusion can be seen on brain MRI in 15–30% of PRES cases, but large homogeneous regions of restricted diffusion like our patient were rare (1, 2). In addition, it is reported that intracranial hemorrhage is common in PRES, complicating 10–25% of cases, especially ongoing therapeutic anticoagulation and intrinsic coagulopathy (1). Bilateral occipital lobe microbleeds in the follow-up MRI may be related to ET.

There are few reports of PRES combined with ET, so the relationship between PRES and ET is not well-understood. Previous studies suggested that endothelial dysfunction may be involved in the mechanism of PRES in ET patients (3, 5). In this patient, the residual contrast agent was still found in the head CT after 3 h of CTA and MRV excluded venous sinus thrombosis, which suggested that there might be venous stasis caused by ET. Venous stasis may lead to PRES by excessive vasodilation and hyperperfusion. In addition, the patient has a stroke-like onset and whether intravenous thrombolysis could be performed if he arrived at the hospital within 4.5 hours. Previous researches showed that there was an increased risk of major bleeding in patients with platelet count >1,000 × 10^9^/L (4). Henrik et al. (6) found that intravenous thrombolysis in thrombocytosis was associated with increased mortality. Therefore, intravenous thrombolysis should be done cautiously for stroke patients with ET history.

The patient had a DNMT3A mutation. Soichi et al. (7) found that *DNMT3A* gene may be associated with myocardial hypertrophy, cardiac dysfunction, and cardiac and renal fibrosis, increasing the risk of cardiovascular disease.

There is no report of triphasic waves in PRES patients so far. The blood ammonia of the patients was 100–125 μmol/L after admission, but the patient had no symptoms of hepatic encephalopathy. The EEG of hepatic encephalopathy mostly showed diffuse triphasic wave changes, which is inconsistent with the focal occipital triphasic waves of the patient (8). In the early stage of onset, the patient developed myoclonic seizure of the left hand, and EEG showed periodic triphasic waves in the bilateral occipital regions, which is similar to the seizure and EEG changes seen in Creutzfeldt–Jakob disease (CJD). Studies have revealed that the EEG patterns are correlated to cerebral imaging findings, and similar EEG changes can occur with different cerebral abnormalities according to the rate of encephalopathy progression, the duration, and so on (9). Therefore, the periodic triphasic waves similar to CJD in this patient may be related to the restricted diffusion of DWI in the cerebral cortex similar to CJD. However, EEG changes in the patient are reversible because of the reversibility of PRES lesions.

Interestingly, the patient experienced hallucinatory palinopsia during the recovery of vision. Visual dysfunction is common in PRES, but there is only one case of palinopsia in PRES patients (10). Palinopsia typically localizes to the non-dominant occipitotemporal cortex, though functional MRI data have suggested its origin in parietal cortical projections to the occipital cortex (11). Its mechanism remains uncertain; cerebral hyperperfusion adjacent to the cortex may be implicated in the pathogenesis (11). The distinctive parieto-occipital pattern and hyperperfusion are the causes of hallucinatory palinopsia in PRES patients.

## Data Availability Statement

The original contributions presented in the study are included in the article/supplementary material, further inquiries can be directed to the corresponding author/s.

## Ethics Statement

The studies involving human participants were reviewed and approved by Peking University First Hospital Ethics Committee. The patients/participants provided their written informed consent to participate in this study. Written informed consent was obtained from the individual's legal guardian/next of kin for the publication of any potentially identifiable images or data included in this article.

## Author Contributions

YL: study concept, data collection, and drafting of the manuscript. YM, MY, YZ, ZL, and ZW: data evaluation and manuscript revision. QP: study concept, data collection, and critical revision. All authors contributed to the article and approved the submitted version.

## Conflict of Interest

The authors declare that the research was conducted in the absence of any commercial or financial relationships that could be construed as a potential conflict of interest.

## Publisher's Note

All claims expressed in this article are solely those of the authors and do not necessarily represent those of their affiliated organizations, or those of the publisher, the editors and the reviewers. Any product that may be evaluated in this article, or claim that may be made by its manufacturer, is not guaranteed or endorsed by the publisher.
